# Willingness to pay for measures to increase use of active school transport

**DOI:** 10.1007/s10198-025-01811-5

**Published:** 2025-07-19

**Authors:** Kristina Ek, Anna-Karin Lindqvist, Ellen Gustavsson Niemi, Stina Rutberg, Nadja Sirviö

**Affiliations:** Luleå University of Technology, Economics Unit, Luleå, Sweden

**Keywords:** Active school transport, Child health, Contingent valuation approach, Health economic, Physical activity

## Abstract

The societal gains from increased physical activity among children are substantial. This study aims to contribute with knowledge about how the benefits of increased physical activity are valued by the Swedish general public. The methodological approach is a contingent valuation survey (*n* = 768), which allows to capture values associated with increased physical activity that extend beyond the health domain. To our knowledge, this is the first study using a contingent valuation approach to assess the comprehensive benefits of promoting physical activity. Although almost all respondents support the idea to promote active school transport in general, slightly more than half the sample reject the idea to contribute financially, while almost half the sample state a positive willingness to pay (WTP), and only approximately 10% state a positive WTP without uncertainty. The average WTP a lump sum for the intervention is estimated to lie between SEK64-185 (approximately 6–19 Euros). Higher WTP correlates with income, perceptions that lack of physical activity as a serious problem, and regular exercise engagement. Due to limited access to cost data, a full economic evaluation is not possible. Nevertheless, even using lower-bound WTP (uncertain = 0), aggregate benefits exceed teacher‐time costs, which are expected to constitute the most important part of intervention costs. Policymakers are recommended to implement low-cost interventions to increase physical activity of children, for instance by promoting the use of active school transport.

## Introduction

The benefits of physical activity (PA) for children are multiple, including improved mental health, bone health, heart health and enhanced academic performance [1–3]. Conversely, insufficient physical activity leads to increased risk of depression, difficulties in concentrating, low energy and sleeping disorders [3, 4]. The recommendation is that children and youths aged 5–17 should engage in 60-minute moderate- to vigorous-intensity physical activity daily [5]. In Sweden, around 85% of girls and 82% of boys aged 11–17 years are insufficiently physically active, closely matching global trends where over 80% of adolescents do not meet WHO recommendations [6]. In the long term, a sedentary lifestyle combined with insufficient PA and unhealthy eating habits increases the risk of developing many diseases such as obesity, cardiovascular disease, type 2 diabetes, and certain types of cancer [7]. Wen and Wu [8] stress that globally, inactivity cause around 5 million deaths annually. Furthermore, Lee et al. [9] report that a 10% reduction in physical inactivity could avert 533,000 deaths per year worldwide, while a reduction by 25% would avert more than 1.3 million deaths. When children engage in healthy habits, it has been shown to generate positive effect also on the behaviour of their parents [10], and active children are also more likely to become active adults [4, 11, 12]. The potential gains for society that can be achieved from the implementation of effective measures to increase the PA of children are thus substantial.

There is a variety of different behavioural, school-based, and incentive-based instruments available to promote PA in children [13]. Given the significant number of children who do not reach the physical activity recommendations, it is vital that interventions have the potential to reach all children. The World Health Organization (WHO) recognizes schools as a potentially important setting for promoting physical activity, as most children spend many hours of the day at school [14]. In this study, we examine how the benefits of an active school transport (AST) project, aimed at school children aged 10–13 in northern Sweden, are valued by the general public. Active travel has shown to be an effective way to increase daily physical activity [15] and it is also important to implement early in life, as it is a predictor for physical activity in midlife [16]. Active travel is also a priority area in the WHO global action plan to increase physical activity [17]. Promoting the use of active transport, walking or cycling, has not only the potential to provide health and cognitive benefits but may also reduce hazardous emissions from traffic and have positive effects on the environment and climate [18–21]. In addition, promoting children’s independent mobility has impact on mental health and personal development [22, 23]. For example, children’s independent mobility may support development of risk assessment skills, orientation skills, self-confidence, and self-esteem [24].

Although the well-documented benefits of regular physical activity on physical, social, and mental health, as well as cognition are widely acknowledged [25], uncertainties persist regarding their magnitude and distribution across individuals, with the most substantial health effects likely occurring in the distant future. The literature assessing the cost-effectiveness of physical activity interventions targeting children is growing. These evaluations typically compare costs with specific outcomes or consequences, such as physical activity levels or health status.^1^ However, previous reviews of these studies have found that effects beyond the health domain are often overlooked. Furthermore, the potential to compare or transfer results from these studies is limited due to methodological heterogeneities such as variation in health metric used and underlying assumptions [26–29].

This study contributes by applying a contingent valuation survey to examine how the general public value the anticipated benefits associated with an intervention aimed at promoting increased use of AST. Respondents are asked about their willingness to pay (WTP) for the intervention, which may include various perceived effects, such as improvements in physical and psychological health, environmental impacts, and the reduction of traffic around schools. Due to lack of cost data, it is not possible to employ a full health economic evaluation. We do however relate estimated WTP to available costs considered to represent significant part of the intervention costs, consisting of salaries for a limited number of teachers that has participated in the implementation of the intervention. Studies using WTP estimates to assess the value of PA interventions targeting children are scarce. One exception is Kesztyüs et al. [30], who analyse parents’ WTP for a program aimed at reducing childhood obesity in Germany through promoting healthy diet, increased physical activity and mindfulness. Another example is Cawley [31] who estimates the valuation of the general public in New York for a similar program by asking about their willingness to accept higher taxes to finance the program. Rome et al. [32] examine WTP for health improvements resulting from physical activity on prescription, targeting adults, in Sweden.

It is worth noting that increased PA may generate benefits beyond those associated with reducing the risk of obesity. In the long run, increased PA in childhood will translate into lower healthcare costs and increased productivity for the whole society. The positive effects will thus accrue both to individuals becoming more active and to society at large, while the direct personal benefits for those financing measures aiming at increasing PA of children are negligible [5]. Consequently, the effects of PA interventions constitute public goods, meaning that individuals solely concerned with their own wellbeing will not have incentives to pay for them. Those who take a stand on the AST intervention may thus be motivated by expectations of future cost savings in the health sector, as well as by concerns for others and the environment.^2^ For example, research has shown that altruistic behaviour positively influences WTP for suicide prevention [33]. Cawley [31] concluded that respondents without children, in particular, may be motivated by altruism (although concern for one´s own children is also a form of altruism).

The purpose of this study is to estimate willingness to pay for increased use of AST in a Swedish context. This knowledge is useful for researchers and for decision makers across various levels. While the societal benefits of children becoming more physically active are potentially substantial, the distribution of these benefits across different domains and over time may pose challenges for the financing of interventions aimed at increasing PA among children. Estimates of how the general public values such interventions can potentially assist decision-makers at various levels in overcoming these challenges.

## Theoretical framework and methodological approach

### The contingent valuation method

The contingent valuation method is a stated preference survey-based method aiming to elicit preferences for non-market goods or services. It enables the estimation of demand for goods or services that are not traded on a market and that lack market prices. In contingent valuation studies, respondents are asked to state their WTP for an increased quantity or quality of the good or service, or the minimum compensation they would require for a decline in its quantity or quality. While contingent valuation is frequently used in environmental and transport economic studies, its application in health economics is less common. The contingent valuation and willingness-to-pay approach was chosen because it allows for multisector effects [34]. Capturing effects beyond the health domain is important in this study, since the AST intervention may have broader impacts, for instance on the environment and traffic safety. Value estimates in contingent valuation studies are based on individual preferences, they are founded in economic theory and can feed directly to measures of utility changes, aiding in prioritising between different alternatives. Contingent valuation studies can thus complement cost-effectiveness studies that compare costs to reach a specific outcome or consequence, such as an increase in Quality-Adjusted Life Years (QALY). However, transferring health effects to monetary units presents ethical challenges, as WTP estimates may reflect income-level differences [35]. For the purposes of our study, where the aim is to estimate the societal benefits of a broad health intervention rather than to determine the use of a specific medical treatment, this limitation is not considered severe. Another limitation of the contingent valuation method is that it is based on hypothetical behaviour; what individuals state may differ from how they act [36]. To reduce such hypothetical biases, the design and careful pre-testing of the questionnaire is crucial. We opted for an open format for the willingness-to-pay question, where respondents state the maximum amount they are willing to pay for the intervention. While a discrete willingness-to-pay question, where individuals are asked if they are willing to pay a certain amount or not, is often considered simpler to answer and may reduce strategic biases [37], we were concerned about anchoring effects, which are known to occur in discrete choice format studies, not least since our pre-tests indicated large variation in individual WTP. In addition, open ended questions provide respondents with the opportunity to express themselves freely and are easy to design.

When determining the payment vehicle, we considered both tax funding and voluntary contributions. School activities are generally tax funded in Sweden, however, the intervention focused on school transport, which is related to school activities but not part of the regular school day. In addition, as the costs for the intervention are expected to be relatively low, and pre-tests indicated that using taxes as a payment format could be perceived as lacking credibility or even provocative, we followed the approach used by Kesztyüs et al. [30]. We chose an open-ended format and asked for a voluntary lump sum donation to help finance the intervention. This approach contrasts with that of Cawley [31], who used a referendum format and asked for the willingness to accept higher taxes.

### Survey design

The intervention used as example in the study aims to increase use of AST [38]. It has been implemented in the recent years in a limited number of schools, targeting children between 11 and 13 years, across three municipalities in different locations in northern Sweden. Rooted in social cognitive theory, the intervention uses empowerment and gamification techniques to engage both students and teachers. The rationale for gamification is that it has potential to make walking and cycling more enjoyable, which can potentially contribute to maintaining these habits also over time. Students are encouraged to track their active travels and solve pedagogical assignments on their way to and from school [39]. Although it is relatively common to use AST in Sweden, it has been gradually declining over the years. In 2006, 62% of children 6–15 years used AST while the corresponding proportion in 2024 was 57%, and this year cycling reached its lowest share since measurement began. Children in Sweden generally have access to bicycles; 9 out of 10 in the ages 6–14 years have a bicycle [40]. In addition, bicycle helmets are mandatory for children under 15 years. Investment costs associated with increased use of AST are thus likely limited. The alternative cost considered most important is the time teachers devote to the intervention, while neither monetary nor time costs are imposed on parents, since children are encouraged to walk or cycle to school without adult or parental company. In spring 2022, seven teachers who had been involved in the intervention, five females and two males, were individually interviewed regarding the time used to its implementation.

The financing and expected benefits associated with the intervention would have widespread societal implication if implemented nationwide. Therefore, the targeted population considered most relevant was the general public rather than solely parents. The first part of the questionnaire aimed to gather data on respondents’ attitudes towards the intervention and their WTP for its nationwide implementation. The WTP-question was preceded by brief information about the intervention and the importance of PA for children. It was mentioned that PA benefits physical and mental health, cognition, and the environment. The questionnaire underwent qualitative pre-testing involving ten individuals and quantitative piloting with 100 respondents. The qualitative pre-tests revealed that a tax as a payment vehicle lacked persuasiveness, leading to the selection of voluntary contributions. The pre-tests also indicated that there were uncertainties about who would receive the funding. Consequently, information was added clarifying that the project was developed by a team of researchers in collaboration with municipal stakeholders, and that funding would support its national scalability. In addition, as a result of the pre-tests, the introductory text was condensed. The result of the pilot showed that a quite high proportion, about half of the respondents, expressed a positive WTP, with WTPs ranging between SEK0–5000 (equivalent to 0-500 Euros). Following the WTP question, respondents were asked to articulate their motives for rejecting or being hesitant to contribute financially. The responses to this question are used to distinguishing between respondents unwilling to pay due to lack of valuation for the intervention (true zeroes) and respondents who refused payment for other reasons, such as rejection of the payment vehicle (protest bids). Responses indicating a general support for the intervention but a refusal to contribute financially, accompanied by agreement with statements suggesting alternative funding methods or the belief that the intervention should be free, were interpreted as protest zero bids.^3^ These responses were excluded from the calculation estimating the average or median WTP. We also calculate a more conservative WTP estimate, based on the positive WTPs after removing protest responses, under the assumption that all uncertain responses are zero. This conservative approach is applied since the voluntary WTP question may lead to overestimated WTP [41]. To assess theoretical validity, the survey also collected socio-economic and demographic information, together with questions about respondents’ attitudes towards physical activity and exercise, as well as their personal habits in this regard. Since WTP was censored at zero we employed a Tobit model when examining the relationship between these socio-demographic and attitudinal variables and WTP [44, 45].^4^ The econometric analysis was conducted in Stata and Nlogit.

## Results

### The sample

A total of 1112 survey responses were collected from a web panel consisting of randomly recruited panellists owned by the company Norstat, in March 2022. Norstat is a data-collecting company that supplies questionnaires to a large panel of 120 000 partially randomly recruited panellists. The participants did not know the topic of the survey before accepting to answer it. Sample descriptives are presented in Table 1. The survey targeted the Swedish population aged between 18 and 80 years. The difference in average ages between the sample and the Swedish population, where respondents in the sample are slightly older than the population in general, is likely a result of distributing the questionnaire to panellists aged 18 to 80, while the population average includes a larger share of younger individuals than those older than 80. The somewhat higher representation of households with children can be explained by the age restriction of the sample, but may also reflect self-selection, as parents may find the topic of the survey more interesting and important than individuals without children and therefore be more prone to finish the survey. Aside from this, there are no substantial differences between the gender and income distributions of the sample and the Swedish population.

**Table 1:** Sample descriptives (*n* = 1112)

	Sample	Swedish population^a^
Age (average)	47	42
Proportion females	0.48	0.50
Proportion with children under 18 in the household	0.29	0.22
Individual gross income per month (SEK) (median and mean) (1 Euro corresponds to approximately SEK10)	30 000–34 999 (interval)	37 100 (mean) 33 200 (median)

^a^ Statistics Sweden: https://www.scb.se/en/

### WTP for the intervention for increased active school transport

**Fig. 1:**
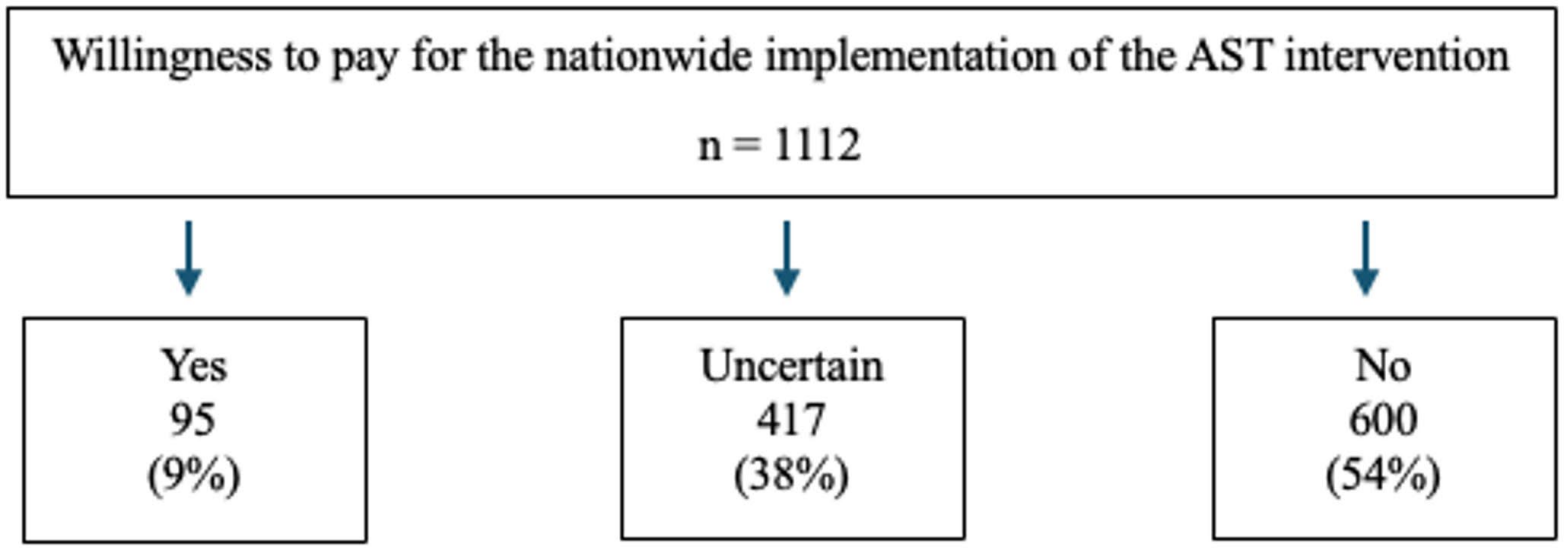
Willingness to pay for the AST intervention

A significant majority, 94% of the total sample, express general support for the implementation of the intervention. The distribution of the responses to the WTP question is illustrated in Fig. 1. Despite the general support, 54% reject the notion of financially supporting the intervention, and additionally 38% indicate uncertainty regarding their financial contribution, while 9% express a willingness to contribute without any hesitation. Those who answered “yes” or “uncertain” were further queried about the maximum amount they would be willing to contribute.

**Fig. 2:**
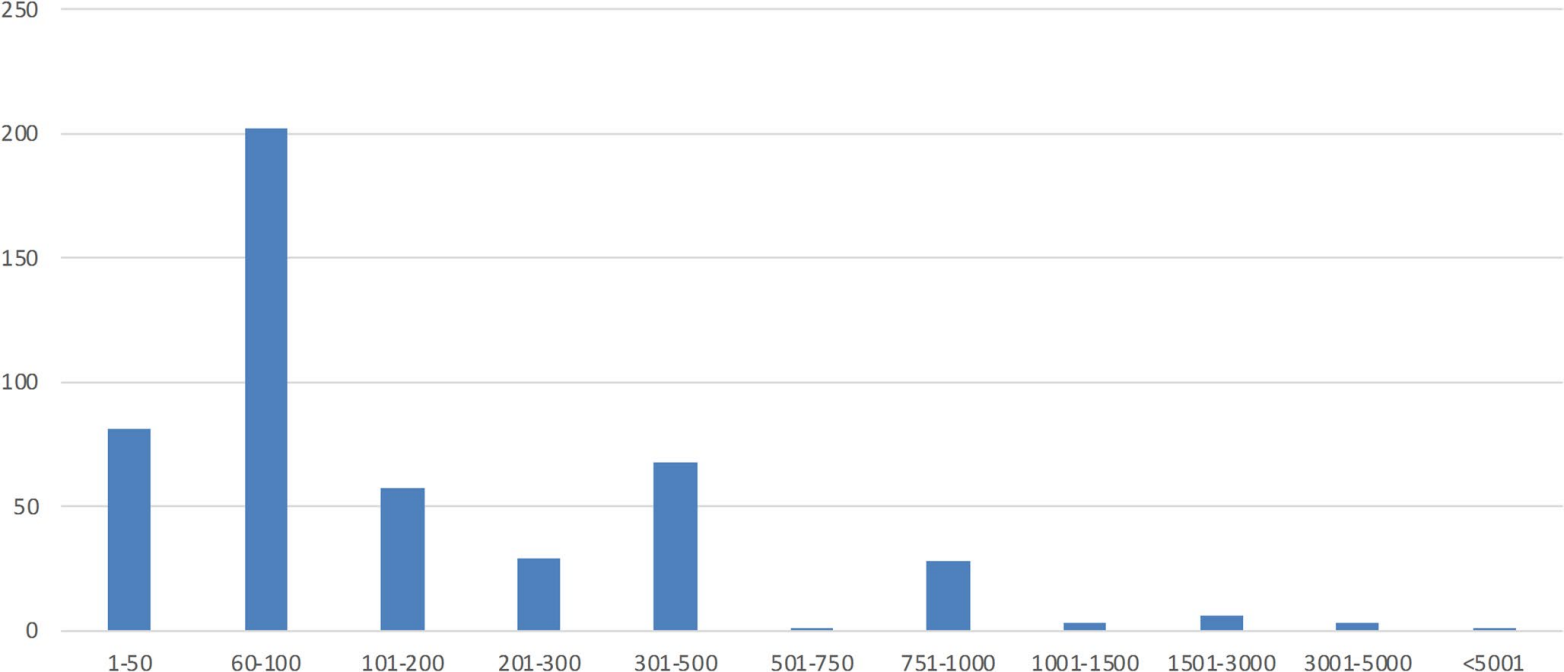
Distribution of positive willingness to pay responses, categories in SEK*. *n* = 769 (protest responses removed, uncertain WTPs included) * SEK10 corresponds to about 1 Euro

Based on the responses on the follow-up question 343 of the 600 rejecting to contribute financially were identified as protest bids.^5^ After excluding these, 769 responses remained for further analysis. After removing protest responses, 297 out of the remaining 769 respondents reported a zero WTP. After applying a binary probit model to identify what socio-demographic characteristics that may be related to the probability to protest, we found female respondents are less likely to be identified as protests, while the probability to protest increase with age and educational level. We found however no statistically significant relationship with the probability to protest and whether there is children in the household or with income level. Figure 2 shows the distribution of the positive WTPs values, clustered in categories. The pattern is similar to the distribution found by Kesztyüs et al. [30], with a notable concentration of WTP values around SEK100. Moreover, a subset of 40 respondents, state a willingness to pay higher amounts, SEK1000 or more.

Table 2 presents average WTP estimates together with standard deviations, minimum, and maximum values for the full sample and three subsamples: the full sample (first row), after removing protests (second row), after removing protests and uncertain WTPs (third row), and after removing protests and assuming that all uncertain positive bids are equal to zero, i.e. the conservative approach (fourth row). The most frequent stated value among the positive bids is SEK100 in all subsamples and the full sample while the median equals zero in all cases except when protest zeroes are removed.

**Table 2:** WTP estimates

	Average WTP (SEK)*	Median WTP (SEK)	Std. dev.	95% C.I. (SEK)	Number of obs.
Full sample	128	0	462	158–212	1112
Protest bids removed	185	100	547	146–224	769
Protest bids and uncertain WTPs removed	141	0	689	68–213	352
Protest bids removed and uncertain WTPs assumed to be zero	64	0	471	31–98	769

* At the time of the study, SEK10 corresponded to about 1 Euro

### Intervention costs and tentative cost-benefit comparison

There are no monetary or time costs imposed on parents, since children are encouraged to independently walk or cycle to school, a practice relatively common in Sweden. Consequently, parents who previously had been driving their kids to and from school will save both time and money. However, since there is no data available on the magnitude of such potential savings, they have not been included in the analysis. Almost all children have access to bicycles and helmets. While the intervention may reduce time costs for parents, it likely results in children spending more time commuting to and from school compared to being driven by car—potentially representing a negative change for some children. However, in the absence of data on transportation times for participating children, this effect cannot be quantified. Furthermore, even if such data were available, assigning a time cost to children’s participation would remain challenging.

The intervention cost is expected to be dominated by the alternative cost of the teachers´ time allocated to the intervention. The responses from the seven teachers that had been involved in the intervention range between 8 and 20 h, with an average and median of 12 h. The median salary of elementary school teachers corresponded at the time of the survey to SEK37900, excluding social fees [42], with an hourly cost, including social fees, of approximately SEK330. Based on these limited time-use estimates, the expected cost for each participating fifth year class amounts to an average cost of about SEK4000. If every one of the 5840 fifth-year classes in the country were to implement the intervention [43], the estimated total cost would amount to nearly SEK23 million.

As indicated by the standard deviations and confidence intervals in Table 2, the WTPs vary substantially, with a high proportion of respondents expressing uncertainty about their willingness to contribute. Consequently, it is cautioned that definitive conclusions about the exact magnitude of WTP should not be drawn. If the most conservative respectively the highest estimated WTPs are aggregated over the population 18 years or older, the total valuation of the program range between a total of SEK0.5 billion for the conservative approach and SEK1.5 billion for the highest estimate of SEK185.^6^ Although these calculations should be interpreted remembering that access to cost data is limited and that the time use estimate is based on very few observations, the aggregated WTP also when based on the most conservative approach exceeds the alternative costs for teachers involved in the intervention.

### Analysing the determinants of willingness to pay

The definitions for the variables included as explanatory variables in the Tobit model are presented in Table 3.

**Table 3:** Variable definitions and descriptives (*n* = 769)

Variable	Definition	Average	Min	Max
Income	Intervals ranging from less than 10 kSek/month to more than 60 kSek/month	30 000–34 999	< 10 kSek	> 60 kSek
Age	Age	45	18	80
Female	Dummy variable equal to 1 if respondent is female, 0 otherwise	0.48	0	1
High education	Dummy variable equal to 1 if respondent has a university education, 0 otherwise	0.36	0	1
Children	Dummy variable equal to 1 if respondent has children under 18 in the household, 0 otherwise.	0.63	0	1
Problem awareness	The extent to which the respondent agrees that physical inactivity is a serious health problem for children, scale ranging between 1–10 (1 representing not at all and 10 in very high degree)	7.9	2	10
Physically active	How often the respondent is moderately to intensely physically active, ranging from 1 (for never) to 7 (for daily)	4.4	1	7

The regression results are presented in Table 4. WTP is positively related to income and negatively related to age and educational level. We find no statistically significant impact on WTP of gender, nor any significant relationship between children in the household and WTP. Respondents reporting that they are concerned about issues stemming from insufficient PA as well as respondents reporting that they are physically active themselves have, on average, higher WTP.

**Table 4:** Tobit regression model results of the amount of willingness to pay

	Coefficient		Standard error	t-value
Income	31.374	***	11.762	2.67
Age	-4.234	**	1.838	-2.30
Female	-4.642		57.592	-0.08
High education	-130.030	**	62.446	-2.08
Children in the household	-64.676		65.646	-0.99
Problem awareness	101.429	***	33.137	3.06
Physically active	49.140	***	17.958	2.74
Constant	-881.748	***	204.022	-4.32

*n* = 769

Log likelihood = -3660

Chi2 = 35.75

## Discussion

Increasing physical activity among children has the potential to bring substantial benefits for both individuals (in terms of physical and mental well-being) and society as a whole (in terms of reduced health care costs, increased productivity, and environmental benefits). However, estimating and comparing the magnitude of these mostly non-priced benefits with intervention costs is challenging, as they span over different domains and long-time horizons. Therefore, there is a significant risk that measures aimed at increasing physical activity, which could be highly profitable for society, are underprovided. Limited information is available to evaluate the cost-effectiveness of interventions for increasing PA, and comparability of previous research is low, due to e.g. differences in outcome measures [27]. This study is the first, to the best of our knowledge, to utilize a contingent valuation approach to examine how measures for increased PA are valued by the general public. The analysis is based on the survey responses from 769 randomly recruited panellists (1112 before protest bids are removed). We use an intervention aiming to increase the use of active school transport as our example. Results indicate strong support in general for the intervention with almost all respondents (94%) expressing support for measures to encourage children to use active school transport. This level of support is slightly lower than the 98% reported by Kesztyüs et al. [30]. We attribute this difference to variation in target populations, as we targeted the general population whereas Kesztyüs et al. [30] collected data from parents in Germany.

However, although almost all respondents support the idea to promote active school transport, when it comes to financing the intervention support is less evident. Slightly less than half the sample indicate that they are potentially willing to contribute financially to the intervention, while slightly more than half reject doing so. Only approximately 10% of the respondents state a willingness to contribute to financing without uncertainty. The discrepancies between stated general support of the intervention and willingness to contribute financially may, at least to some extent, be related to the payment vehicle. The responses to the follow-up questions indicate that relatively large proportions of respondents support the intervention but refuse to contribute financially. This may be the result of protest bids, for instance, beliefs that the level and frequency of children´s physical activity should not require payment, or that the intervention should be funded by the tax system. Additionally, the discrepancies may result from free-rider behaviour, where respondents place value on the intervention but expect others to bear the financial burden.

The estimated average WTP values range between €13–19 (corresponding to SEK64-185) and are lower than those reported by Kesztyüs et al. [30], who estimated an average WTP of €23 (corresponding to about SEK230) and significantly lower than the USD46 reported by Cawley [31] (corresponding to about SEK460). However, the studies of Cawley [31] and Kesztyüs et al. [30] focus specifically on obesity prevention whereas our focus is on the valuation of increased PA of children, considering all the different benefits it could generate. While the contingent valuation allows for considering multisector effects, it is not possible to disentangle the relative importance of different perceived impacts on WTP responses. It is likely that the relative importance of health versus environmental benefits varies among respondents.

The analysis of correlates of WTP did not raise validity concerns; a higher willingness to pay was found among respondents with higher income (see e.g. Cawley [31]; Kesztyüs et al. [30]; Romé et al. [32]). Respondents who exhibit problem awareness demonstrate a significantly higher WTP on average compared to those who are less concerned, a finding consistent with the result of Cawley [31]. Problem awareness emerges as the most influential factor, having the largest impact on WTP. Respondents engaging in at least moderate PA frequently are also willing to pay higher amounts than people who are not exercising regularly. We find this result reasonable, as we could expect individuals who are engaged in physical activities and training to also be more attentive in issues related also to public health and healthy behaviour. We did not have specific a priori expectations regarding the relationship between age and WTP. Kesztyüs et al. [30] did not include age in the analysis of correlates of WTP, while Cawley [31] reported a positive relationship. Our results indicate that older respondents have on average lower WTP than younger respondents. We expected respondents with children in the household to be more willing to allocate income for the intervention, since they might expect their household to benefit from it. We did however not find statistically significant evidence to support such a relationship.

Since additional costs aside from the alternative cost for teachers involved in implementing the AST intervention are expected to be limited, we believe the intervention has potential to be attractive from a cost perspective. Nevertheless, a more thorough cost analysis based on e.g. time use of a larger number of teachers as well as changed time use for school transports by parents and children is called for. This is an area for important future research.

Although we have no serious indications that the sample is not representative of the population, it is still possible that respondents who are particularly engaged in issues related to physical activity are overrepresented in our sample. Since this group has a higher willingness to pay than people less engaged in this topic, there is a risk that the willingness to pay is overstated. Another limitation worth remembering is the implication of the hypothetical setting and the non-incentivized WTP question. When involving voluntary contributions to a public good primarily associated with non-use values, the WTPs should possibly be interpreted within the contribution model proposed by Kahneman and Knetsch [46], rather than within a traditional welfare economic purchase model. According to Kahneman and Knetsch, the latter is more appropriate for public goods that also generate use-values. Nevertheless, even with this somewhat softer interpretation, and using the conservative WTP estimate (acknowledging that a vast majority are rejecting or hesitant to financing the intervention), implies that significant values are associated with measures aimed to deal with the insufficient PA of children.

## Conclusion

The results indicate that a large majority of the respondents state that they support an intervention aimed at increasing the use active of school transport among children. When it comes to contributing financially, results are however more mixed. About half of the sample are potentially willing to contribute financially, and approximately 10% are willing to do so without uncertainty. Still, when aggregating over the lowest WTP estimate, assuming that all uncertain respondents have zero WTP, estimated benefits are significantly higher than the alternative cost for teachers involved in the intervention, which are expected to constitute the most important part of intervention costs. Based on these results, policymakers on various levels are recommended to implement low-cost interventions to increase physical activity of children.

## Footnotes

1 Most of these are cost-effectiveness studies focusing on obesity prevention, in which the outcome, in terms of improved health related quality of life (often measured in QALYs) or reduced risk for deteriorating health as a result of overweight and obesity (measured in DALYs), can be compared with intervention costs (see e.g. Breheny et al. [47] Formatting.; Moodie et al. [48] Formatting.; Moodie et al. [49] Formatting.

2 Altruistic preferences can – at least in theory - be separated into pure altruism (the utility of an individual is affected by the utility of someone else), impure altruism (the utility of the individual increases when she/he thinks she/he did a good deed) and paternalistic altruism (the utility of an individual depends on an argument of the utility function of someone else). It is however beyond the scope of this paper to examine this further; we note though that altruism may play a role for the decision whether to contribute financially or not.

3 Respondents supporting the intervention in general, refusing to contribute to funding and agreeing with statements saying, “I do not think it is something I should have to pay for” or “I think the intervention should be financed in another way” were identified as protest responses. Responses refusing to pay and agreeing with the statements “I cannot afford”, “I do not think measures for increased physical activity are needed”, “I need more information before I take a stand”, “I think it is the responsibility of the parents to promote physical activity”, or “I am not interested” were considered true zeroes.

4 The Cragg’s Double Hurdle Model was also considered. It can be appropriate in situations where households first decide whether to participate or not and secondly decide how much they will pay, Cragg’s specification allows the modelling of these two decisions in two steps [50]. In a log likelihood-ratio test of whether the Cragg’s model should be favoured over the Tobit specification, we could not reject the Tobit model in favor of the Cragg specification and the Tobit model was preferred.

5 198 respondents agreed with the statement “I do not think it is something I should have to pay for” while 270 agreed with “I think the intervention should be financed in another way”. It was possible to agree with more than one of the alternative statements in the follow-up question, which explains that these statements sum to more than 343.

6 According to Statistics Sweden (scb.se), there were 8.3 million inhabitants 18 years or older in the country at the time of the study while the total population amounted to approximately 10.5 million inhabitants.

## References

[1] Majeed, S.: Role of physical activity and sports in mental health of youth: A review Article. Shield Res. J. Phys. Educ. Sport Sci. **17**, 1–20 (2022)

[2] Käll, L.B., Nilsson, M., Lindén, T.: The impact of a physical activity intervention program on academic achievement in a Swedish elementary school setting. J. Sch. Health. **84**(8), 473–480 (2014) 25040115 10.1111/josh.12179

[3] World Health Organization: WHO guidelines on physical activity and sedentary behaviour: Accessed 24 June 2024. (2020) 33369898

[4] Gc, V.S., Suhrcke, M., Turner, D., Atkin, A.J., Van Sluijs, E.: Cost-effectiveness of physical activity interventions in adolescents: Model development and illustration using two exemplar interventions. BMJ Open. **9**(8) (2019). 10.1136/bmjopen-2018-027566 10.1136/bmjopen-2018-027566 PMC6701678 31427318

[5] World Health Organization: Global recommendations on physical activity for health. Geneva: WHO::8–10. (2010). https://www.who.int/publications/i/item/9789241599979. Accessed 24 June 2024 26180873

[6] Guthold, R., Stevens, G.A., Riley, L.M., Bull, F.C.: Global trends in insufficient physical activity among adolescents: A pooled analysis of 298 population-based surveys with 1·6 million participants. Lancet Child. Adolesc. Health. **4**(1), 23–35 (2020). 10.1016/S2352-4642(19)30323-2 31761562 10.1016/S2352-4642(19)30323-2 PMC6919336

[7] Gaetano, A.: Relationship between physical inactivity and effects on individual health status. J. Phys. Educ. Sport. **16**, 1069–1074 (2016). 10.7752/jpes.2016.s2170

[8] Wen, C.P., Wu, X.: Stressing harms of physical inactivity to promote exercise. Lancet. **380**(9838), 192–193 (2012). 10.1016/S0140-6736(12)60954-4 22818933 10.1016/S0140-6736(12)60954-4

[9] Lee, I., Shiroma, E.J., Lobelo, F., Puska, P., Blair, S.N., Katzmarzyk, P.T.: Effect of physical inactivity on major non-communicable diseases worldwide: An analysis of burden of disease and life expectancy. Lancet. **380**(9838), 219–229 (2012). 10.1016/S0140-6736(12)61031-9 22818936 10.1016/S0140-6736(12)61031-9 PMC3645500

[10] Berniell, L., de la Mata, D., Valdés, N.: Spillovers of health education at school on parents’ physical activity. Health Econ. **22**(9), 1004–1020 (2013). 10.1002/hec.2958 23780620 10.1002/hec.2958

[11] Rauner, A., Jekauc, D., Mess, F., Schmidt, S., Woll, A.: Tracking physical activity in different settings from late childhood to early adulthood in germany: The MoMo longitudinal study. BMC Public. Health. **15**(1), 1–11 (2015). 10.1186/s12889-015-1731-4 25887314 10.1186/s12889-015-1731-4 PMC4407713

[12] Telama, R., Yang, X., Leskinen, E., et al.: Tracking of physical activity from early childhood through youth into adulthood. Med. Sci. Sports Exerc. **46**(5), 955–962 (2014) 24121247 10.1249/MSS.0000000000000181

[13] Ikeda, E., Hinckson, E., Witten, K., Smith, M.: Assessment of direct and indirect associations between children active school travel and environmental, household and child factors using structural equation modelling. Int. J. Behav. Nutri Phys. Act. **16**(1), 1–17 (2019). 10.1186/s12966-019-0794-5 10.1186/s12966-019-0794-5 PMC6451289 30953526

[14] World Health Organization: Promoting physical activity in the education sector: (2018). https://www.euro.who.int/en/health-topics/disease-prevention/physical-activity/publications/2018/promoting-physical-activity-in-the-education-sector-2018 Accessed 24 June 2024

[15] Williams, G.C., Borghese, M.M., Janssen, I.: Neighborhood walkability and objectively measured active transportation among 10–13 year olds. J. Transp. Health. **8**, 202–209 (2018). 10.1016/j.jth.2017.12.006

[16] Yang, X., Telama, R., Hirvensalo, M., Tammelin, T., Viikari, J.S.A., Raitakari, O.T.: Active commuting from youth to adulthood and as a predictor of physical activity in early midlife: The young Finns study. Prev. Med. **59**, 5–11 (2014). 10.1016/j.ypmed.2013.10.019 24201092 10.1016/j.ypmed.2013.10.019

[17] World Health Organization: Global action plan on physical activity 2018–2030: More active people for a healthier world: (2018). https://apps.who.int/iris/bitstream/handle/10665/272722/9789241514187-eng.pdf. Accessed 24 June 2024

[18] Larouche, R., Saunders, T.J., Colley, R., Tremblay, M., Faulkner, G.E.J.: Associations between active school transport and physical activity, body composition, and cardiovascular fitness: A systematic review of 68 studies. J. Phys. Act. Health. **11**(1), 206–227 (2014). 10.1123/jpah.2011-0345 23250273 10.1123/jpah.2011-0345

[19] Hartog, J.J., Boogaard, H., Nijland, H., Hoek, G.: Do the health benefits of cycling outweigh the risks? / Os benefícios à Saúde Em Andar de bicicleta superam Os riscos? Ciência. Saúde Coletiva. **16**(12), 4731–4744 (2011). 10.1590/S1413-81232011001300022 10.1590/s1413-81232011001300022 22124913

[20] Mueller, N., Rojas-Rueda, D., Cole-Hunter, T., et al.: Health impact assessment of active transportation: A systematic review. Prev. Med. **76**, 103–114 (2015). 10.1016/j.ypmed.2015.04.010 25900805 10.1016/j.ypmed.2015.04.010

[21] Pérez, K., Olabarria, M., Rojas-Rueda, D., Santamariña-Rubio, E., Borrell, C., Nieuwenhuijsen, M.: The health and economic benefits of active transport policies in Barcelona. J. Transp. Health. **4**, 316–324 (2017). 10.1016/j.jth.2017.01.001

[22] Campos-Sanchez, S.F., Abarca-Alvarez, F.J., Molina-Garcia, J., Chillon, P.: A GIS-based method for analysing the association between school-built environment and home-school route measures with active commuting to school in urban children and adolescents. Int. J. Environ. Res. Public. Health. **17**(7) (2020). 10.3390/ijerph17072295 10.3390/ijerph17072295 PMC7177458 32235341

[23] Riazi, N.A., Brussoni, M., Vertinsky, P., Faulkner, G.: Well, you feel more responsible when you’re unsupervised: Exploring family perspectives on children’s independent mobility. Children-Basel. **8**(3) (2021). 10.3390/children8030225 10.3390/children8030225 PMC7998357 33804287

[24] Riazi, N.A., Faulkner, G.: Children’s independent mobility. In: Children’s Active Transportation.:77–91. (2018). 10.1016/B978-0-12-811931-0.00005-3

[25] Kohl, H.W., Craig, C.L., Lambert, E.V., et al.: The pandemic of physical inactivity: Global action for public health. Lancet. **380**(9838), 294–305 (2012). 10.1016/S0140-6736(12)60898-8 22818941 10.1016/S0140-6736(12)60898-8

[26] Döring, N., Mayer, S., Rasmussen, F., Sonntag, D.: Economic evaluation of obesity prevention in early childhood: Methods, limitations and recommendations. Int. J. Environ. Res. Public. Health. **13**(9), 911 (2016). 10.3390/ijerph13090911 27649218 10.3390/ijerph13090911 PMC5036744

[27] Korber, K.: Quality assessment of economic evaluations of health promotion programs for children and adolescents-a systematic review using the example of physical activity. Health Econ. Rev. **5** (2015). 10.1186/s13561-015-0071-5 10.1186/s13561-015-0071-5 PMC4658341 26603159

[28] Lehnert, T., Sonntag, D., Konnopka, A., Riedel-Heller, S., Knig, H.H.: The long-term cost-effectiveness of obesity prevention interventions: Systematic literature review. Obes. Rev. **13**(6), 537. (2012) 10.1111/j.1467-789X.2011.00980.x 22251231

[29] Zanganeh, M., Adab, P., Li, B., et al.: A systematic review of methods, study quality, and results of economic evaluation for childhood and adolescent obesity intervention. Int. J. Environ. Res. Public. Health. **16**(3), 485 (2019). 10.3390/ijerph16030485 30743995 10.3390/ijerph16030485 PMC6388206

[30] Kesztyüs, D., Lauer, R., Schreiber, A.C., Steinacker, J.M., Kesztyüs, T., Kilian, R.: Parents’ willingness to pay for the prevention of childhood overweight and obesity. Health Econ. Rev. **4**(1), 8 (2014). 10.1186/s13561-014-0020-8 26208923 10.1186/s13561-014-0020-8 PMC4883987

[31] Cawley, J.: Contingent valuation analysis of willingness to pay to reduce childhood obesity. Econ. Hum. Biology. **6**(2), 281–292 (2008). 10.1016/j.ehb.2008.05.003 10.1016/j.ehb.2008.05.003 18619930

[32] Rome, Å., Persson, U., Ekdahl, C., Gard, G.: Willingness to pay for health improvements of physical activity on prescription. Scand. J. Public. Health. **38**(2), 151–159 (2010). 10.1177/1403494810364559 20064920 10.1177/1403494809357099

[33] Vimefall, E., Persson, M., Sara, P.D., Hultkrantz, O.: Is prevention of suicide worth less? A comparison of the value per statistical life. Eur. J. Health Econ. **23**(2), 261–275 (2022). 10.1007/s10198-021-01361-6 34420119 10.1007/s10198-021-01361-6 PMC8882109

[34] Olsen, J.A., Smith, R.D.: Theory versus practice: A review of `Willingness-to-pay’ in health and health care. Health Econ. -Chichester. **10**(1), 39–52 (2001). 10.1002/1099-1050 10.1002/1099-1050(200101)10:1<39::aid-hec563>3.0.co;2-e 11180568

[35] Frew, E.: Aligning health economics methods to fit with the changing world of public health. Appl. Health Econ. Health Policy. **3**, 287 (2017). 10.1007/s40258-017-0318-2 10.1007/s40258-017-0319-9 28258395

[36] Blumenschein, K., Blomquist, G.C., Johannesson, M., Freeman, P.: Eliciting willingness to pay without bias: Evidence from a field experiment. Econ. J. **118**(525), 114–137 (2008). 10.1111/j.1468-0297.2007.02112.x

[37] Perman, R.: In: Harlow (ed.) Natural Resource and Environmental economics / Roger Perman, 4 edn. Pearson Addison Weslay, United Kingdom 415–429 (2011). https://research-ebsco-com.proxy.lib.ltu.se/linkprocessor/plink?id=89d736ea-d130-308f-96ef-dffe3f01112c

[38] Trafikverket. Attitydundersökning barns skolvägar 2024: (2025). https://trafikverket.diva-portal.org/smash/get/diva2:1944332/FULLTEXT01.pdf

[39] Lindqvist, A., Rutberg, S.: One step forward: Development of a program promoting active school transportation. JMIR Res. Protoc. **7**(5) (2018). 10.2196/resprot.9505 10.2196/resprot.9505 PMC5964300 29739733

[40] Swedish Transport Administration: National cycling accounts 2023: How is cycling developing in Sweden? 2024. Available from: https://trafikverket.diva-portal.org/smash/get/diva2:1895596/FULLTEXT01.pdf

[41] Liljas, B., Blumenschein, K.: On hypothetical bias and calibration in cost-benefit studies. Health Policy. **52**(1), 53–70 (2000). 10.1007/s10995-015-1898-4 10899644 10.1016/s0168-8510(00)00067-1

[42] Swedish Association of: Local Authorities and Regions. Facts about salaries. (2022)

[43] Swedish National Agency for Education: Information about school units, study paths and statistics. (2022)

[44] Amore, M.D., Murtinu, S.: Tobit models in strategy research: Critical issues and applications. Global Strategy J. **11**(3), 331–355 (2021). 10.1002/gsj.1363

[45] Donaldson, C., Jones, A.M., Mapp, T.J., Olson, J.A.: Limited dependent variables in willingness to pay studies: Applications in health care. Appl. Econ. **30**(5), 667–677 (1998). 10.1080/000368498325651

[46] Kahneman, D., Knetsch, J.L.: Valuing public goods: The purchase of moral satisfaction. J. Environ. Econ. Manage. **22**(1), 57–70 (1992). 10.1016/0095-0696(92)90019-S

[47] Breheny, K., Adab, P., Martin, J., et al.: Effectiveness and cost-effectiveness of the daily Mile on childhood weight outcomes and wellbeing: A cluster randomised controlled trial. Int. J. Obes. **44**(4), 812–822 (2020). 10.1038/s41366-019-0511-0 10.1038/s41366-019-0511-0 PMC7101281 31988481

[48] Moodie, M., Haby, M., Galvin, L., Swinburn, B., Carter, R.: Cost-effectiveness of active transport for primary school children-walking school bus program. Int. J. Behav. Nutri Phys. Act. **6**(1), 63 (2009) 10.1186/1479-5868-6-63 PMC2758827 19747402

[49] Moodie, M.L., Herbert, J.K., de Silva-Sanigorski, A.M., et al.: The cost-effectiveness of a successful community-based obesity prevention program: The be active eat well program. Obesity. **21**(10), 2072 (2013). 10.1002/oby.20472 23554382 10.1002/oby.20472

[50] Cragg, J.G.: Some statistical models for limited dependent variables with application to the demand for durable goods. Econometrica. **39**(5), 829–844 (1971). 10.2307/1909582

